# Metal-Based Nanoparticles in Food Packaging and Coating Technologies: A Review

**DOI:** 10.3390/biom13071092

**Published:** 2023-07-07

**Authors:** Jerry O. Adeyemi, Olaniyi A. Fawole

**Affiliations:** Postharvest and Agroprocessing Research Centre, Department of Botany and Plant Biotechnology, University of Johannesburg, P.O. Box 524, Auckland Park, Johannesburg 2006, South Africa

**Keywords:** food security, nanoparticles, food packaging, biopolymers, biological properties

## Abstract

Food security has continued to be a topic of interest in our world due to the increasing demand for food. Many technologies have been adopted to enhance food supply and narrow the demand gap. Thus, the attempt to use nanotechnology to improve food security and increase supply has emerged due to the severe shortcomings of conventional technologies, which have made them insufficient to cater to the continuous demand for food products. Hence, nanoparticles have been identified to play a major role in areas involving food production, protection, and shelf-life extensions. Specifically, metal-based nanoparticles have been singled out to play an important role in manufacturing materials with outstanding properties, which can help increase the shelf-life of different food materials. The physicochemical and biological properties of metal-based nanoparticles, such as the large surface area and antimicrobial properties, have made them suitable and adequately useful, not just as a regular packaging material but as a functional material upon incorporation into biopolymer matrices. These, amongst many other reasons, have led to their wide synthesis and applications, even though their methods of preparation and risk evaluation remain a topic of concern. This review, therefore, briefly explores the available synthetic methods, physicochemical properties, roles, and biological properties of metal-based nanoparticles for food packaging. Furthermore, the associated limitations, alongside quality and safety considerations, of these materials were summarily explored. Although this area of research continues to garner attention, this review showed that metal-based nanoparticles possess great potential to be a leading material for food packaging if the problem of migration and toxicity can be effectively modulated.

## 1. Introduction

Food security in developing countries faces challenges such as low agricultural productivity, inadequate farming practices, natural resource degradation, high post-farming losses, limited value addition, and rapid population growth. Many methods are thus being adopted with newer technologies such as genetic modification, methods for improving soil fertility, biofortification, synthetic biology, artificial intelligence, and irrigation technologies to enhance food supply and narrow the demand gap, according to reports made by the United Nations Conference on Trade and Development (UNCTAD) in 2017 [1].

The attempt to use nanotechnology for agricultural purposes has been thought to emerge from the inferences that conventional farming technologies are insufficient to increase the ever-growing need for productivity while maintaining an eco-friendly approach [2]. For instance, the long-term use of “miracle seeds” with other farming techniques and agents such as pesticides, fertilizers, and irrigation has been questioned at the scientific and policy levels and proposed to be phased out due to the many health and environmental concerns [2]. Furthermore, foodborne illnesses are also a global public health concern. The Centers for Disease Control (CDC) estimated 47.8 million foodborne diseases, 127,839 hospitalizations, and 3037 deaths in 2011 in the United States alone [3], which were thought to result in productivity losses and severe economic consequences. This data has also culminated in the increasing demand for new technologies to control foodborne pathogens, which has significantly increased in recent years. A significant amount of postharvest losses for fruits and vegetables are associated with plant pathogens of fungal origins [4]. Due to high nutrient and moisture content, and low pH, fruits and many vegetables are prone to pathogenic fungi attacks which cause rots, producing mycotoxin, making them unfit for consumption [4]. Although estimating postharvest losses through rots and decay is not easy to calculate due to differences in commodity, seasons, and production area, the losses are very significant, especially in under-developing tropical countries, which conservatives’ estimations have placed at 50% for perishable commodities [4]. As such, food packaging plays a vital role in providing safety and maintaining food quality.

Years ago, most manufacturers, including food manufacturers, focused mainly on new trends in food packaging [5]. These materials were primarily designed and formulated only to protect the product. Its increasing importance in marketing became a significant factor in winning consumers [5]. However, these days, they have been carefully designed to retard product deterioration, extend shelf-life, maintain processing benefits, and manage the quality and safety of food items [6]. Thus, in recent times, food packaging has been carefully designed to protect from various external influences; physical, chemical, and biological. Therefore, these secondary functions highlight their role in maintaining food quality and preventing foodborne diseases. Hence, food packaging with new functions is termed active packaging [7]. They are developed due to consumer demand for safer and more natural products with a longer shelf life, better cost–benefits, and convenience [7]. Based on the European Union regulations, active packaging is a material that possesses the ability to change the properties of the food or the atmosphere around it when in contact [8]. Antimicrobial packaging is an example of active packaging that interacts with the product to retard, inhibit or reduce the growth of microorganisms on food surfaces [9]. These materials allow for the easy and intermittent diffusion of antimicrobial agents into food matrixes, eliminating the need for further addition of antimicrobials directly to the food product [7]. Hence, active packaging for food materials can play an additional role in reducing the risk of food pathogens and extending their shelf life. Many materials of pure organic origins, such as enzymes, organic acids, bacteriocins, and essential oils, have been considered as polymeric matrices in the production of active packaging materials with the potential to exert antimicrobial properties on food materials [4,10,11,12]. However, their sensitivities to industrial conditions, such as high pressure and temperature, and the development of microbial resistance have limited their usage [7]. This has thus led to increasing interest in the use of nanotechnology as an alternative or add-on option in the quest to find suitable material for food packaging [7].

Generally, Nanotechnology has wide applications in multiple areas. It has been the subject of considerable research and studies due to the possibility of controlling a single atom or molecule to achieve the formulations of different types of new materials for specific usage [13]. Recent advancements in nanotechnology have revolutionized several fields like medicine, pharmaceuticals, renewable energy, and agricultural sciences [2,14]. Nanotechnology has already shown promising solutions in improving agricultural productivity and reducing losses via nano fertilizers, herbicides, pesticides, soil feature regulation, wastewater management, and pathogen detection [14]. This technology, likewise, has benefited industrial food processing sectors with enhanced food production, excellent market value, high nutritional and sensing properties, improved safety, and better antimicrobial protection [14]. This field is an advanced emerging research area in which matter is altered to a particular size in nanoscale between 1–100 [15], with the smallest unit termed nanoparticles. Particles with sizes found in this range are mainly composed of metal, metal oxides, organic matter, and carbon [13]. Nanotechnology thus bridges the physicochemical gap between atoms/molecules and bulk (macroscopic) material [16]. 

Many reports have already established the superior properties of materials at the nanoscale than their bulk counterparts [16]. The observed dissimilarity between these materials has been attributed to the small sizes and high surface area-to-volume ratio of nanoparticles [16]. The high aspect ratio is also, among many other reasons. Specifically, inorganic (metal-based) nanoparticles have shown excellent antimicrobial properties even at low concentrations due to their unique physicochemical properties [17]. They are stable in extreme conditions, such as high temperatures and pressure, and some constituting metal elements of these nanoparticles are essential minerals for the human body. This, in turn, make some of them biocompatible with human cells, nontoxic, and a suitable choice of material [18].

Metal-based nanoparticles have attracted extensive attention as an alternative/additive material in this regard owing to their excellent mechanical properties, barrier capabilities, biocompatibility, and broad-spectrum antimicrobial performances [19]. These nanoparticles have been widely employed as active additives in biomedical and food technology. Most metal-based nanoparticles such as Ag, Au, ZnO, CuO, and TiO_2_ [20,21,22] possess good antioxidating and antimicrobial properties, which makes them attractive in the design of novel materials in the agricultural sector. For instance, several reports exist on the synthesis and biological usefulness of Ag, Au, and their bimetallic nanoparticles [23,24,25,26]. Furthermore, ZnO is one of the most synthesized and studied nanoparticles due to its exceptional antioxidative and antimicrobial properties [27,28,29]. Zinc oxide nanoparticles have specifically been studied widely due to the ease of preparation, safety, and acceptance by the U.S. Food and Drug Administration (21CFR182.8991) (FDA, 2011) [30]. All these properties and applications have culminated in the continuous interest in metal-based nanoparticles in many biomedical fields, agriculture, and food packaging industries [28,31].

This review aimed to provide a comprehensive understanding of the role of nanotechnology, specifically focusing on metal-based nanoparticles, in food packaging applications. This will be achieved by highlighting various synthesis methods used to produce metal-based nanoparticles, as well as the physicochemical and biological properties that render them suitable for use in food packaging production. Additionally, the unique characteristics that make these nanoparticles valuable in the development of active food packaging materials will be explored. While discussing their advantages, the potential limitations and concerns associated with their implementation in food packaging will also be addressed. Due to the presence of certain identified constraints, this review will solely concentrate on metal-based nanoparticles that have already been legislated and approved for use in the field.

### Types of Nanomaterial for Food Packaging

Food packaging has remained an essential component of the food production process. Nevertheless, permeability has continued to be a challenge for most conventional packaging materials as none is entirely water and atmospheric-resistant [32]. This problem, in turn, has led to the seeking of alternative solutions that are innovative, cost-effective, environmentally friendly, safe, durable, and capable of monitoring packaged food quality [33]. Therefore, many factors, such as transportation, handling, and environmental contamination protection, have been reported to drive innovative discoveries of food packaging materials [34,35]. All these are influenced by the continuous demands from consumers for high-quality and nutritious food products [36,37]. The use of non-biodegradable substances like plastics, glass, and metals has continued to garner serious environmental concerns. Consequently, other materials like nanobiocomposites are being developed with the capacity for other functions that can enhance the shelf life while reducing the negative environmental impacts [33]. Hence, many enhanced food packaging, resulting from an upgrade to the basic functions of traditional packaging materials, designed to improve food quality, safety, and shelf life while also providing additional information have been developed and termed by many names such as smart, active, intelligent, interactive, clever, responsive, and diagnostic materials [38]. Nevertheless, intelligent packaging (IP) materials precisely have the capacity to monitor the environment inside or near the packaging and react appropriately, while active packaging (AP) offers increased food protection. However, the concept of smart packaging combines the benefits of both active and intelligent packaging technology [39].

The different nanomaterial that has been applied thus far in food packaging can be generally classified as organic or inorganic. The organic materials include whey proteins, polysaccharides, quaternary ammonium salts, chitins, halogenated compounds, and phenols [40,41]. On the other hand, inorganic nanomaterials are often metal-based, which are further categorized into pure metals, metal oxides, and metal and/or metal oxide composites [42,43]. These are incorporated into compositing materials, usually polymers, to make nanocomposite films and nanofibers [42,43]. Hence, many organic, inorganic, and composite nanomaterials have been effectively developed for the qualitative and quantitative losses of food materials. Some prominent examples that have been successfully applied in food packaging are nanocellulose, nanostarch, protein nanoparticles, chitosan nanoparticles (CNPs), carbon nanotubes, silver nanoparticles (Ag-NPs), nanoclay, zinc oxide nanoparticles (ZnO-NPs), titanium oxide (TiO_2_-NPs) [44].

## 2. General Synthetic Approaches for the Preparation of Nanomaterials

**Figure 1:**
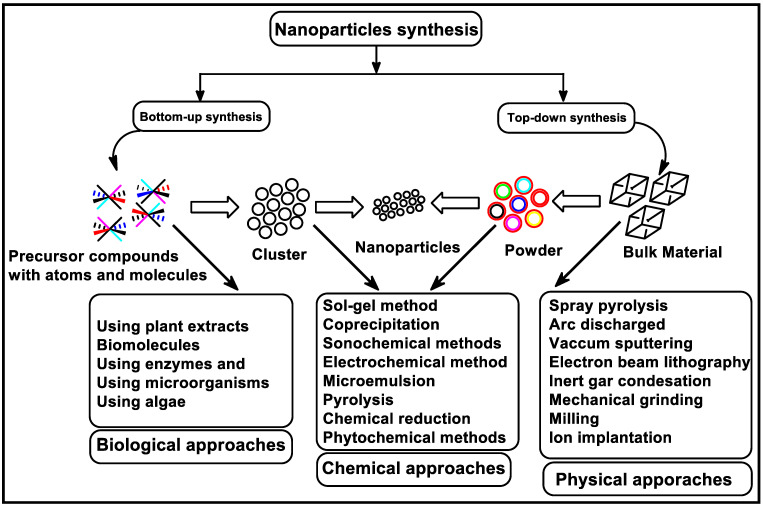
Different synthetic methods of nanoparticles. Image copied and modified from [50], with permission from Elsevier (Copyright 2023).

Detailed synthetic approaches have already been well established in literature over the years. These methods are generally categorized as “top-down” or “bottom-up” approaches [45,46,47]. The top-down method involves size reduction from a starting material via different types of physical or chemical treatment [48]. However, this method produces materials with limited surface chemistry and physical properties due to the introduction of imperfections on the surface of the material [16]. In bottom-up methods, the nanomaterials are built up from small particles like atoms to form a new entity in the nano regime [49]. In this approach, the nanostructured building blocks of the nanoparticles are formed as the first step and then assembled to produce the final particle [16]. Methods under the bottom-up approach are primarily chemical and biological reliant. Figure 1 summarizes various synthetic methods under the “top-down” or “bottom-up” approaches.

Based on the projected application of choice, these different synthetic approaches have been employed in synthesizing material with unique and exciting characteristics [51]. Nevertheless, the associated toxicity accompanying most of these methods has been a major environmental concern in recent years because of toxic organic solvents, reducing substances, and stabilizers. The waste and ecological concerns material has led to the desire for a more biologically compatible, clean, reliable, efficient, and environmentally friendly synthetic route, such as using plant extracts or microorganisms [45]. These biological methods are generally referred to as the biogenic/green method. A detailed review of the biogenic synthesis and mechanisms involved in the preparation of metal-based nanoparticles has already been published and reviewed [52,53].

## 3. Metal-Based Nanoparticles in Food Packaging Technology

Food packaging materials of nanomaterial origin for shelf-life extension and quality retention are generally synthesized majorly by incorporating nanoparticles, which may be derived from either metal or metal oxide, into conventional food packaging materials such as films or containers, composite multilayer materials, organic, inorganic, and combined coating material [54]. Nanotechnology thus helps produce materials with improved properties like enhanced physical and mechanical properties while also preferring solutions to food deterioration by exhibiting biological properties such as antibacterial, antioxidative, and UV absorption properties [44]. Furthermore, these materials also perform a smart packaging property by actively monitoring and controlling food conditions within the enclosed package [55]. Some currently used nano-based food packaging materials that have already gained acceptance and are commercially used have been summarized in Table 1. As projected from the statistical report of the Vantage Market Research, the global smart packaging market size is projected to reach an estimated $33 billion by 2028, with a compound annual growth rate (CAGR) of 12% during the forecast period from the year 2021 till 2028 [56]. Further projection placed the smart packaging market as the fastest expanding material in the coming years and has alluded to its growth rate to its unique, interactive, customer-friendly features at a less expense [44].

### 3.1. The Physicochemical Properties, Roles, and Biological Properties of Metal-Based Nanoparticles and Their Relevance in Food Packaging

The food packaging industry has become an important sector in food production due to the emergence of new technologies for retaining the nutritional and organoleptic properties of stored food [44]. Hence, in recent time food packaging scope has gone further than conventional food preservation to include the preservation of sensitive bioactive compounds from unfriendly environmental and physical damages [44]. This new scope has, in turn, led to the extensive search for material with functional properties such as thermal strength, stability, durability, and improved barrier properties, which possess the capacity to extend shelf- life of food products [54,57]. It is noteworthy to state that, generally, food packaging material should be made from cheap, hard, flexible, lightweight, inert, and strong sources, amongst other useful properties [58]. Two notable materials that fall into this class are polypropylene and polyethylene. Nevertheless, these materials are non-biodegradable because they are plastic-based materials that may take more than a hundred years to break down and non-recyclable [58], which thus constitutes environmental hazards. Consequently, other solutions that are sustainable, biodegradable, safe, and can prevent or reduce environmental concerns are currently being sought [58]. Hence, materials that do not pose health or environmental concerns and are easily disposed of are the most desired. Natural products from renewable materials of animal, plant, and other biological origins are used [58]. Nevertheless, despite the benefits of natural biopolymers in food packaging application, they do not have the optimal required barrier, physical, and mechanical properties [58]. This, therefore, has brought about the introduction of nanomaterials with relevant properties capable of improving the currently conventional available food packaging material. Nano-based packaging materials for shelf-life extension and quality retention have thus been majorly synthesized either by the incorporations of nanoparticles into some already available traditional food packaging materials, which include films and containers or through the design of new nanocomposite with multi-layered materials and inorganic, organic, or the combination of both by nanocoating through, spraying, rubbing and immersion [44].

Over the years, metal-based nanoparticles have been extensively studied because of their benign nature and outstanding physical, biological, and physicochemical properties. Their ease of preparation also accounts for the numerous documentation in literature [26,52]. The optical properties, which are mainly influenced by the localized surface plasmon resonance (LSPR) of the noble metal nanoparticles, are significant properties that make this class of valuable nanomaterial in sensing and, ultimately, as smart packaging material [59,60]. This property has also allowed their use as material in drugs and gene delivery, photothermal therapy, molecular labeling, and bioimaging [61]. Furthermore, their ease of bioconjugation and low toxicity made them highly suitable and sought after for various biological studies (antimicrobial, anti-inflammatory, antiviral, anti-platelet, antidiabetic, anti-angiogenesis, and anticancer agents) and bio-nanotechnology [62].

Metal-based nanoparticles are, therefore, of particular interest in nanotechnological research for food packaging materials [58]. This is because they can be easily incorporated into natural biopolymers to form hybrid materials called nano-biocomposite, which possess significantly better properties than their respective ones. Metal-based nanoparticles have been reported to actively participate in the efficient design of active, novel, and efficient packaging [63,64]. For instance, loading varied from 0 to 5 wt% of ZnO Nps on a glycerol plasticized-pea starch film and the use of carboxymethyl cellulose as a stabilizer has been reported by Yu et al. [65]. This report showed an increase in the mechanical strength (tensile) by 9.81 MPa and 42% elongation at break, according to the report by Yu et al. [65]. Furthermore, higher UV-visible absorption properties were observed for this material. Therefore, the loading of nanoparticles on natural biopolymers often results in the formation of active packaging, which often performs other roles other than the conventional packaging material. Table 2 gives a summary of metal-based nanoparticle and their respective role in the design of some active food packaging materials using some natural biopolymers [58].

### 3.2. Prominent Examples of Metal-Based Nanoparticle and Their Food Packaging Applications

Silver nanoparticles (AgNP) have remained at the forefront of the most studied metal-based nanoparticles due to their unique physical and chemical properties, which have led to their application in several fields of endeavor [52]. Over the years, silver has shown to be a valuable material for food protection against microorganisms in the production of liquid food substances such as wine, water, and milk [33]. Its application in medicine and biotechnology remains amongst its notable applications due to its ability to inhibit the growth of microorganisms attacking humans, such as those in burns, catheters, cuts, and wounds, to protect them from infection [76]. Due to its large surface area, silver, in its nano form, has been reported to possess a broad spectrum of biological activities such as antimicrobial, antifungal, anti-yeasts, antioxidant, and antiviral compared to its bulk counterpart [77,78,79]. The two forms of silver, Ag^0^ and Ag^+^ species, have been suggested to account for the antagonistic action of silver nanoparticles (AgNPs) against microorganisms [37]. Furthermore, they have been reported to have the capacity to break down lipo-polysaccharide by binding to the surface of the cell [33]. Hence, there has been extensive interest in studying their different synthetic routes.

In the past, silver NPs have been prepared using conventional methods such as the solvothermal synthetic route, requiring many hazardous, pricey, and environmentally unfriendly chemicals [80]. These concerns have led to the discovery of many more accessible, easy-to-prepare, cheap, and ecologically friendly approaches, such as using plant extracts as a mediating agent. Although using biologically significant extracts in synthesizing these nanomaterials have been found to confer enhanced bioactivity on these materials; nevertheless, the conventional methods have been reported to possess the potential to control the shape of the nanoparticles more readily. However, toxic chemicals, cost, and wastes, which influence their biocompatibility, remain a significant concern [10]. Many plants such as Musa *balbisiana* (banana), *Azadirachta indica* (neem) and *Ocimum tenuiflorum* (black tulsi), *Phyllanthus emblica*, *Dovyalis caffra, Clitoria ternatea*, *Solanum nigrum*, and *Jasminum officinal* have been used in the preparation of silver nanoparticles with different biological properties [52,81,82,83].

**Figure 2:**
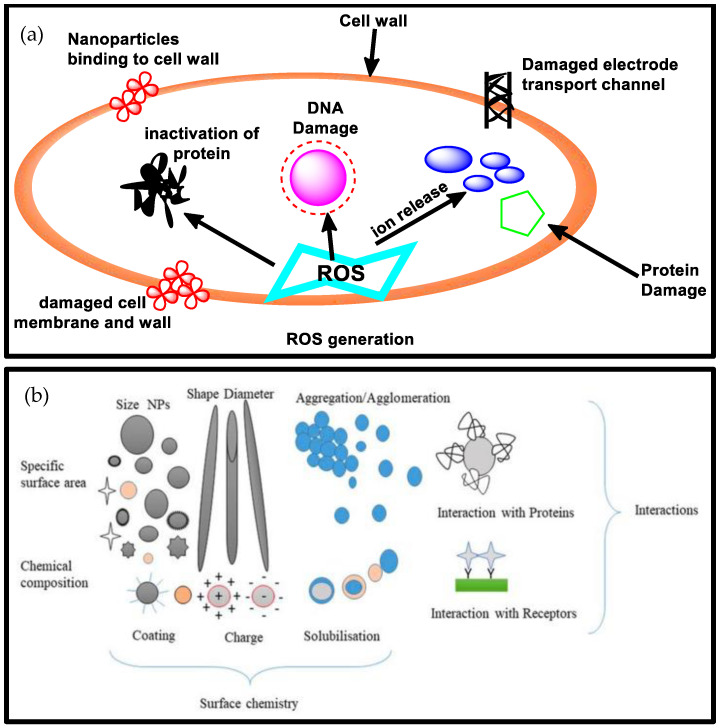
(**a**) mechanism of antimicrobial action [44] and (**b**) the impact of some physicochemical properties on the interaction of metal-based nanoparticles with microbial cells (images copied from [96] with permission from Elsevier (Copyright 2023).

Silver has been one of the most explored in the class of metal-based nanoparticles due to its already established acts as an antimicrobial agent against several commensal and pathogenic strains alongside fungi and viruses [84,85]. They act by targeting metabolic activities through their binding to DNA, proteins, and enzymes, which results in bacteriostatic effects [86]. This then disrupts and destabilizes the outer and cytoplasmic membranes [87]. They have also been found to stimulate the production of reactive oxygen species (ROS) and inhibit some enzymes responsible for the respiratory chain, as seen in Figure 2 [88]. The influence of physiochemical properties such as shape, size and crystal structure, as seen in Figure 2b, on the antimicrobial activities of metal-based NPs is well established in literature [89,90]. Nevertheless, other factors such as aggregation, dissolution and surface charges have been implicated in the biological activities of these materials. The dissolution process, for instance, is a crucial process in which the nanoparticles release metal ions, which can interact with bacterial cells, disrupting their vital functions and leading to cell death [91]. This enhances the antibacterial activity of metal nanoparticles and contributes to their effectiveness against Gram-positive and Gram-negative bacteria [92]. Although agglomeration has been dubbed to enact both positive and negative effects on the performance of metal-based nanoparticles against microorganism, its impact on the activities have been highlighted in literature. Agglomerated nanoparticles can increase surface area for interactions with bacterial cells due to the larger structure provided by the aggregated material, allowing for increased contact and interaction with bacterial membranes [92]. Nevertheless, the large, agglomerated structures may also limit the penetration of nanoparticles into bacterial cells, reducing their effectiveness while still altering the physicochemical properties of the nanoparticles, such as size, shape, and surface charge, which may affect their interaction with bacteria and their mode of action [92]. The surface charges of nanoparticles have been thought to significantly influence antimicrobial activities. The surface charges of nanoparticles, such as silver nanoparticles, affect their interactions with bacteria and contribute to their antibacterial properties [93,94,95]. However, the exact mechanisms underlying these interactions and the specific effects of nanoparticle surface charges on antimicrobial activities require further research and exploration [93,94,95].

Silver nanoparticles have been embedded in porous zeolite, which is used in producing plastic (low-density PE) with the capacity to extend the shelf life for storing beverages such as orange juice [88,97]. The active nanocomposite has been reported to be highly effective in conferring antimicrobial properties and possessing heat treatment capacity [88,97]. Most biopolymers prepared uses polysaccharides and protein for food packaging. Many studies have been carried out in which nanoparticles like silver have been impregnated into polymeric matrixes. For instance, about 15.3 mgmL^–1^ of silver NPs impregnated into cellulosic food packages was found to significantly enhance the shelf life of tomatoes and cabbage, according to the report by Singh and Sahareen [98]. Also, Vieira et al. used about 0.25% (*w*/*w*) Ag-NPs to inhibit the proliferation of *Colletotrichum gloeosporioides* on stored fruits (*Carica papaya* L.) for 14 days at 20 °C, thus extending the shelf life [97]. Likewise, using PVP as a coating material, 72 and 98 mgmL^–1^ of silver nanoparticles were used to inhibit the growth of *E. coli* and *Bacillus cereus*, respectively, which in turn prevented the development of grey molds for 15 days at 15 °C on stored pepper chilli [99]. Similarly, fresh stored tomatoes’ shelf life has been extended by 30 days using 100 mgmL^–1^ of Ag NPs compared to the used control, according to [100]. Other studies using silver nanoparticles as food packaging materials and their notable properties have been summarized and presented in Table 3.

Zinc oxide nanoparticles, just like silver NPs, have remained at the forefront of oxides nanoparticles that have received considerable attention due to their attractive physicochemical, cost, and role in many biological systems because of the presence of Zn, which is an essential element for both plants and animals [100]. Zinc oxide and silver NPs have been extensively studied and used as antibacterial, anti-inflammatory, antifungal, antioxidant, cancer therapy, wound healing, bioimaging, antidiabetic, drug delivery, and drug targeting purposes [106]. In its oxide form, Zn has been reported to become readily available for assimilation compared to its ionic form. It was found during a foliar study of coffee plants that the Zn content of the plant was three times more in the plant in which ZnO was used compared to the ionic form, which in turn led to the high assimilation of carbon dioxide and high photosynthetic rate [107]. This thus highlights the importance of using the oxide than the ionic form. It has been reported that using ZnO NPs on the surface of plant material and upon exposure to light generates reactive oxygen species such as H_2_O_2_, *OH, and O^2^*–). These active oxygen species attack the microbial wall of microorganisms, inhibiting their proliferation and growth on such plant material. Hence, reducing or completely stopping the spoilage of perishable food materials [7,108]. Nevertheless, ROS also tends to impede the homeostasis of many plant systems [109]. There is, therefore, a need to find the right balance in its postharvest application to serve the desired purpose and not generate a new problem entirely. Moreover, the U.S. FDA has acknowledged ZnO as a “generally recognized as safe” (GRAS) material (21CFR182.8991) (FDA,2011) [7]. Due to the broad-spectrum potential against several microorganisms, ZnO has been used in food packaging in compositing with other polymeric materials. The most common material matrix used with ZnO is Chitosan [110,111,112,113]. In a study carried out to compare the antimicrobial performance of Ag-Chitosan film against ZnO-Chitosan counterpart, using *S. aureus*, *E. coli*, *S. typhamrium*, *B. cereus*, and *L. monocyte* ranged, the inhibition diameter for silver was between 10–15 mm while ZnO was between 15–19 mm. In another study involving the screening of an incorporated ZnO NPs in a matrix mixture of chitosan/calcium silicate/polyethylene glycol against *S. aureus*, *P. aeruginosa*, *C. albicans,* and *A. nigera* a higher inhibition diameter was reported which was superior to those of the control used in the study [114]. The incorporation of chitosan into other biopolymers has also been extensively studied. For instance, incorporating ZnO into polypyrrole-modified bacterial cellulose as a polymer matrix, a material currently used in food packaging applications, revealed a remarkable improvement as an antioxidant material [115]. Furthermore, the nanorod of ZnO has been composited with grapeseed extract to form a film that showed UV-blocking and enhanced vapor barrier properties [116]. Other studies in which ZnO has been incorporated into polymeric matrices are summarized in Table 4.

Another notable metal-based nanomaterial that has been considered and is currently being investigated is titanium oxide nanoparticles (TiO_2_ NPs) [122]. This is because, alongside ZnO and silver, they have been approved as safe materials by The Food and Drug Administration (FDA) for biomedical, food, and cosmetics applications [123,124]. Specifically, TiO_2_ NPs, within the size range of 20–400 nm, have been widely used as packaging material in the food industry because of their biocompatibility, non-toxicity, high surface area, UV absorptivity, high refractive index, photocatalytic, and biological properties [125,126,127]. Titanium oxide has already been approved as a food additive in many countries, including the USA. However, the stipulated amount by the FDA has been limited to 1% of the total food mass. Contrary to the USA, the EU has approved titanium oxide use to quantum satisfaction, meaning no maximum level is specified [122]. These laws, therefore, show the suitability of the material in food packaging and an approved food additive (listed as E 171) in quantum satisfaction, which means that no maximum level is specified [128]. In China, up to 10 g/kg of TiO_2_ can be used in food substances as a coloring agent [128]. In medicine, food, cosmetics, and electronics, TiO_2_ NPs have been widely used due to their valuable properties [129,130]. It has been specifically used in the food sector to manufacture active packaging composite film with improved functional properties [127,131]. Titanium oxide interacts with the film matrixes, which in turn leads to enhanced physical strengths, improved gas barrier, and, in some cases when confers a secondary function of decomposing ethylene, which in turn leads to enhances the shelf life of fruits after harvest [125,127,129,132].

Hence, its outstanding properties, such as ethylene scavenging abilities [133,134], antimicrobial properties [135,136,137], compatibility with biopolymers [131,138], and UV shielding [136,139] have exceptionally made them useful in the design of active food packaging materials [127]. Upon its addition to biopolymers in the preparation of composite films, these properties are generally enhanced [132,134]. This also leads to the concurrent enhancement of the physical, barrier, mechanical, thermal, functional, and chemical properties of the polymeric matrix [131,137,138,140]. These properties are measured based on solubility, thickness, and moisture content for physical properties; water vapor and oxygen for barrier properties; color coordinates and transparency for optical properties; glass transition temperature (Tg), melting point, and degradation temperature for thermal properties; tensile strength, elongation at break, Young’s modulus for mechanical properties; gas scavenging, antioxidant, antimicrobial, and UV shielding, for functional properties [127]. The functional properties of biopolymers containing titanium oxide used in active food packaging material have been widely studied and summarized in Table 5 [122].

Also, copper oxide is among the notable FDA-approved metal-based nanoparticles that have garnered attention. Their potential as antimicrobial agents (such as bacteria, fungi, viruses, and algae) has made them highly desirable for several applications [155]. This is because the high surface area of the nanoparticles allows for interaction with cell membranes, which confers an excellent antimicrobial action [156,157,158]. Furthermore, the increased interest stems from the observed properties such as shape, size, and composition [159] and outstanding physical properties like high-temperature superconductivity, electron correlation effects, and spin dynamics [160]. This has led to its application in several scientific and technological fields, including electronics [161,162], agriculture [163,164], medicine [165,166], and solar energy [167,168]. Its function as an antimicrobial agent stems from the fact that copper ion destroys and interrupts microbial cell components by redox reactions. Their antimicrobial potential has thus been highly studied and applied in improving some polymers used in food packaging [157,158,169,170]. In a study carried out by Saravanakumar et al. [171] to produce an antimicrobial film (APF), CuO NPs have been incorporated into cellulose at different compositions using sodium alginate (SA) as a plasticizer to provide flexibility. Both constituent materials, cellulose nano-whiskers (CNW) and the CuO NPs, acted synergistically by limiting moisture penetration and preventing microbial activity on freshly cut pepper. In this study, the standard characterization technique of XRD, UV, FTIR, EDX, and SEM was carried out to ascertain the resulting physicochemical properties of the newly prepared material. This material was found to exact the active food packaging of antimicrobial and barrier actions at the optimum compositions of CNW (0.5%)-SA (3%)-CuO NPs (5 mM), which showed the potential to be a functional food packaging material capable of overcoming the limitations of the conventional ones [171]. The prospects of two or more FDA-approved metal-based nanoparticles have been studied to examine the possibility of synergism in their application as food packaging materials. According to the report made by Dehghani et al. [172], some FDA-approved metal nanoparticles of Ag, ZnO and CuO at different combination ratios at a reduced concentration were incorporated into LDPE to prepare an active food packaging material. The physicochemical characterization confirmed uniformly distributed nanoparticles on the surfaces of the prepared nanocomposites. It was found that at a combination of up to 1% (*w*/*w*) of any two NPs, improved tensile strength and elongation at break properties of the films were observed. Furthermore, in some specific combinations containing ZnO NPs, the UV transmission was reduced, which means they possess the potential to prevent the adverse effect of UV deterioration. Against *Staphylococcus aureus* and *Escherichia coli*, these materials showed increased antimicrobial action in the various combinations without increasing concentrations. It was concluded that the LDPE without the combination involving Ag (i.e., ZnO-CuO combination) showed the best food packaging potentials regarding strength and antimicrobial actions. This showed the potential of the combination of metal-based nanoparticles than the individual ones seeing that enhanced activities were recorded [172].

### 3.3. Practical Application of Metal-Based Nanoparticles Composites to Food Materials

As already established in many studies carried out in literature, which showed the potential of metal-based nanoparticles in improving the properties of food packaging materials, many studies have already applied these materials to real-life food substances such as fruits, oils, and meats. For instance, upon embedding ZnO nanoparticles at varying compositions into CMC-based functional films with grape seed extract to high-fat beef and investigating for 15 days, the number of psychotropic bacteria in the composite coating contains 3% ZnO film was within the acceptable range of 5.9 Log CFU/g. Additionally, it was observed that the same composite film with the 3% ZnO prevented the oxidation of lipid in the meat upon refrigeration (reducing by 88%), which thus suggest that this material could be useful as an active packaging material for high-fat meat such as beef [120].

**Figure 3:**
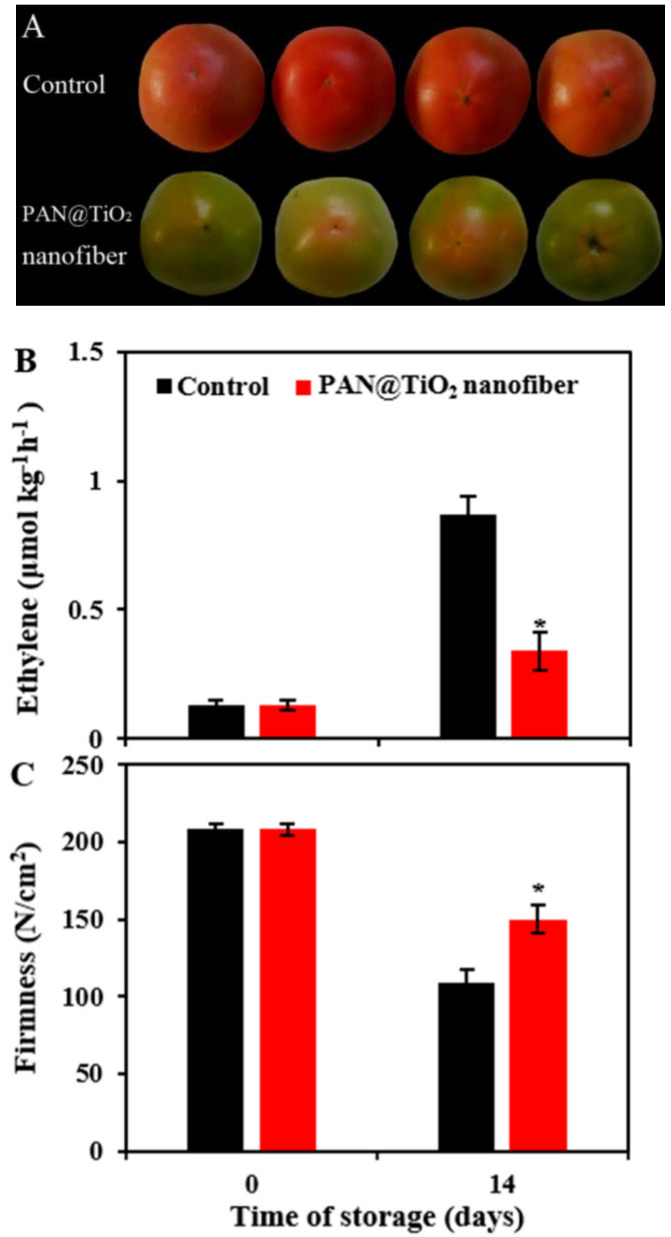
Images showing the effect of ethylene production (**A**), the data of the impact of coating with PAN@TiO_2_ nanofibers. On ethylene production (**B**) and firmness (**C**) for tomatoes stored for 14 days. The error bars show the standard deviation, while the * indicates significant differences between nanofiber-covered fruit and the control based on Student’s *t*-test (*p* < 0.05). Copied with permission from MDPI (Copyright 2023) [173].

In the fruit industry, a notable concern plaguing the preservation of fruits for longer storage is the problem relating to the generation of hormones that enhances the natural ageing and decaying of perishable foods like fruits and vegetables during postharvest storage and transportation. Thus, removing this from the surrounding environment can significantly improve their shelf life and reduce the damage to food materials [173]. In this study by Zhang et al., TiO_2_ nanoparticles composited alongside polyacrylonitrile (PAN) were examined for the degradation potential of fruit-emitted ethylene using photocatalysis [173]. The prepared PAN@TiO2 composite showed enhanced photocatalytic activity in ethylene degradation under low-intensity UV light irradiation (2.9 µWcm^−2^), which in turn slowed the color change and the softening of the tomatoes during storage for 14 days (see Figure 3) [173]. Approximately 65% of the ethylene was degraded within 25 h. The report thus showed the potential of TiO_2_-coated PAN nanofibers as a valuable material for shelf-life extension for food materials such as tomatoes [173]. It was noted alongside other literature that photocatalytic processes can remove acetaldehyde, ethanol, and off-flavours generated by red tomatoes during storage [174].

Furthermore, the shelf life of fresh-cut food has been reported to be significantly reduced from several weeks to days due to the various metabolic activities on the tissues of the fruits, which include damages during grating, peeling, and shredding; and the exposure of the cut surfaces to external surroundings [175]. Thus, modifying the environment around the food could offer a solution for shelf-life extension by adjusting the barrier properties of the packaging film [176]. Edible coatings like those in which metal-based nanoparticles are embedded have been documented to show a promising approach to this problem. Li et al. [175] have used PVC film with ZnO nanoparticles to examine the shelf-life extension effects on freshly cut ‘Fuji’ apples at 4 °C for 12 days. It was observed that, upon comparing with the ordinary PVC film, the fruit decay was significantly reduced alongside the accumulation of malondialdehyde (MDA) from 74.9 nmol/g in the control to 53.9 nmol/g in the nano-packaging [175]. Although the cutting process was reported to bring about the increased generation of ethylene, suggesting wound-induced ethylene production, this was significantly suppressed in the fruit packaged with the ZnO composites. Additionally, it was found that both the pyrogallol peroxidase and polyphenol oxidase activities were also decreased in the prepared nanocomposite. The initial appearance of apple slices was retained, and the browning index was prevented in nano-packaging samples.

Other studies using ZnO nanoparticles alongside polysaccharides as safe coating materials and their respective activities are summarized in Table 6 [177].

According to many reports in literature, silver nanoparticles (AgNPs) have already proven to be one of the most effective antimicrobial nano-based materials with a broad-spectrum activity against different microbial pathogens, including bacteria, fungi, yeasts, and viruses [184,185,186]. This has made them one of the most sought-after nanomaterials in material science. Hence, they have been composited with many polymeric materials including biopolymers and plastic materials for various food packaging applications. In this study by Kumar et al., [186] packaging film of Ag nanoparticles-based nanocomposite with both chitosan and gelatin bases were formulated. This report showed that, at varying composition of 0.05% and 0.1%, the addition of Ag nanoparticles (obtained from a green synthetic route using plant extract of fresh *Mimusops elengi* fruit) to the polymer matrixes led to an enhanced mechanical property and decrease in light transmittance in the visible light region. Its application on red grapes gave an extension of shelf life by a fourteen day period. In another report by Kowsalya et al., [185] electrospun silver nanoparticles/poly(vinyl alcohol) composite was prepared by incorporating the synthesized Ag nanoparticles (10% (*w*/*v*) PVA and 0.5% (*w*/*v*) of Ag), which was also prepared using plant extracts of *Vitis vinifera* (black grapes), in poly(vinyl alcohol) matrix, for fruits preservation. This material showed a good antimicrobial action against different food pathogens upon coating on lemon and strawberry, extending the shelf life and preventing the decay for up to 10 days.

Like silver, Cu-based nanomaterials are highly sort after due to their biological potentials. Although few, most of the application in which Cu-based nanoparticles have been employed in literature as food packaging material involves those in which antimicrobial action is imposed on the material, mainly in nonbiodegradable plastic matrices and few biopolymers [187]. For instance, the shelf-life elongation of freshly cut yellow bell pepper has been examined using composites of CuO nanoparticles and cellulose/SA-based biodegradable polymer, according to Saravanakumar et al. [171]. This coating was reported to significantly reduce the propagation of bacterial growth (*Salmonella* spp. and *Listeria* spp.) while lowering the total fungi count in the bell peppers for seven days. Furthermore, in another report, CuO nanoparticles embedded in a bilayer pouch for the preservation of coconut oil were found to reduce the oxidation of coconut oil for over three months [188]. Nevertheless, prolonged usage may be a health risk due to potential bioaccumulation, even though the films are made of edible coating materials. Other reports in which CuO nanoparticles incorporated into methylcellulose films have been applied in food material, including their usage as material for prolonged shelf-life extension of hard cheese [160], which resulted in the inhibition of microbial growth during storage at 35 °C for one week.

## 4. Limitation of Nanotechnology in Food Packaging

As the application of nanoparticles in food, drug, and cosmetics continues to grow, several agencies like the FDA, IFAS, and USEPA have started considering the potential risk of using nanoparticles in general in different products [189]. For instance, the FDA 2006 has initiated a task force to determine the human, animal, and plant risks of using this class of materials which seems to be gaining serious attention in research. Furthermore, the environmental impact and the sources of the nanomaterial have also been of concern [190]. Consequently, the FDA, and other international bodies, like the EU, have provided adequate information and guidance to evaluate the safe use of nanoparticles in food packaging alongside standardized procedures to analyze the risk to humans and the environment [191].

Many concerns have been raised over the years regarding the continuous usage of nanomaterial in the area involving food and drug due to concerns around toxicity and bioaccumulation [192]. As the application of nanoparticles in food, drug, and cosmetics continues to grow, several agencies like the FDA, IFAS, and USEPA have started considering the potential risk of using nanoparticles in general in different products [189]. For instance, the FDA 2006 has initiated a task force to determine the human, animal, and plant risks of using this class of materials which seems to be gaining severe attention as years go by. Their environmental impact and the sources of the nanomaterial have also been of concern [190]. One concern in the area of toxicity, which has raised many unanswered questions over the years concerning the use of nanomaterials in food substances, generally, especially in the design of novel food packaging materials, is the migration of harmful components into food [44]. Over the past few years, extensive research has focused on the migration of nanoparticles into food substances. Silver nanoparticles have received significant attention due to government concerns regarding their safety and health implications. These studies have revealed that nanomaterials can enter the body through various pathways, leading to their distribution across different organs. Moreover, they can adversely affect human cells by altering mitochondrial function, generating reactive oxygen species, enhancing membrane permeability, and inducing toxic effects. As a result, nanoparticles such as silver have been implicated in the development of chronic diseases, including allergies, asthma, inflammations, cardiovascular disorders, and cancer [193]. Some studies have already attributed the toxicity brought about via migration in food substances through the large-surface-area-to-volume ratio of these nanomaterials [194]. Nevertheless, toxicity has been thought to vary depending on factors such as time of exposure, the concentration of material, and individual reactivity [195].

Generally, the migration process of nanomaterials in food packaging can be divided into two stages. The initial stage of migration occurs when nanomaterials encapsulated within the surface layers of the packaging material are released. The subsequent stage involves the release of nanomaterials from the interior part of the packaging, which must pass through voids and gaps between the polymer molecules [196]. The extent and speed of this migration process depend on various factors. The migration of nanomaterials into food depends on the chemical and physical properties of the food and the polymer used in the packaging. Factors such as the initial concentration of nanomaterials, particle size, molecular weight, solubility, and diffusivity of the specific substance in the polymer, as well as pH value, temperature, polymer structure and viscosity, mechanical stress, contact time, and food composition, are the main parameters that control the migration process [197]. Studies have shown that the encapsulated nanomaterials inside the film may sometimes need to oxidize and migrate out through the polymer matrices. These encapsulated nanomaterials are primarily responsible for the release of nanomaterials at later times. The solubility of metallic nanoparticles in aqueous solutions increases with higher temperatures and lower pH values, which can lead to an increased migration of metals in the system [198].

Therefore, identifying and characterizing nanomaterials in the food are necessary due to the potential risks they pose to consumers. the ability of the nanomaterials to migrate from food packaging to the food itself makes it crucial to employ specific techniques to evaluate and analyze these materials [193,197]. To accurately measure nanomaterials in complex matrices, it is essential to use analysis techniques that can distinguish between nanoparticles and other components present. Furthermore, these techniques should be sensitive enough to detect low concentrations of nanomaterials while providing sufficient information about their concentration, composition, and physicochemical properties within samples. However, the determination of their exact quantity of food materials is currently impossible. Nevertheless, in such a complex situation, and at this moment, the amount of migrated nanoparticles in food, synthetic methods are necessary to determine the quantity of migrated nanoparticles and detect them, as independent methods cannot provide all the required information [193,199]. As such, conventional chromatography methods are limited and unsuitable for analyzing polymer additives, as they cannot measure the physicochemical properties of nanoparticles. Consequently, only a few methods effectively detect nanoparticles and determine their properties. These include Microscopic Methods, Quantitative Analysis Methods, and Spectroscopy Methods [193,200].

Nanostructured materials exhibit characteristics that can help kill bacteria, such as generating reactive oxygen species (ROS), the release of heavy metal ions, or increasing the specific hydrophobic surface area. However, these same characteristics can also lead to cytotoxicity or debilitation of mammalian cells [95]. Hence, one notable concern that must be considered when using metal-based nanoparticles is the issue regarding dissolution, especially when exposed to biological molecules such as thiols [89]. Dissolution is an important characteristic that affects the bio-durability and persistence of nanoparticles, which may be employed to predict the possible environmental or health effect [201]. Biomolecules can influence the dissolution of metal-based nanoparticles, and this interaction has implications for human health. This action thus proceeds when metallic ions, in the presence of an aqueous medium, slowly get discharged from their oxides, followed by absorption by the cell membranes, which then leads to interaction with nucleic acids and proteins. This consequently brings about variations and aberrant enzymatic actions, which ultimately disturb the expected physiological properties of the cell [89]. Thiol-containing molecules can interact with the surface of nanoparticles, forming bonds between the metal atoms and thiol groups. This interaction can either enhance or inhibit the dissolution of nanoparticles depending on various factors such as the specific metal, nanoparticle properties, and environmental conditions [201]. The dissolution process of ZnO, for instance, often involves the release of Zn^2+^ ions into the surrounding medium, which is usually influenced by various factors, including pH, temperature, and the presence of biomolecules. Specifically, Wang et al. investigated the interaction of ZnO nanoparticles with thiol-containing molecules, such as glutathione (GSH) or cysteine [202]. It was observed that the biomolecules interacted with the surface of the nanoparticles to form stable complexes via Zn-S bonds, slowing down the dissolution process and effectively inhibiting the release of Zn^2+^ ions by forming a surface passivation layer [202]. Similarly, thiol groups from molecules like cysteine or mercaptoundecanoic acid can bind to the surface of Ag nanoparticles, forming Ag-S complexes. This interaction can either passivate the surface and reduce dissolution or, under certain conditions, enhance the dissolution of Ag nanoparticles [203,204]. Likewise, l-cysteine or glutathione have been reported to retard the dissolution rate of TiO_2_ by forming surface complexes which in turn affects its dissolution kinetics [205].

The consequences of metal-based nanoparticle dissolution for human health depend on various factors, including the type of metal, the concentration of metal ions released, and the route and duration of exposure. Metal ions released from nanoparticles can interact with biological systems and potentially induce adverse effects. Some metal ions, such as cadmium, lead, and mercury, are toxic to humans, even at low concentrations. Their presence in the body can disrupt cellular processes, cause oxidative stress, and lead to various health problems [201]. It is important to note that the behavior of metal-based nanoparticles and their interaction with biomolecules is still a complex area of research, and the specific outcomes for human health can vary depending on the nanoparticle characteristics, exposure conditions, and the specific metal involved. Further studies are needed to understand the mechanisms and potential risks associated with the dissolution of metal-based nanoparticles in the presence of biomolecules.

Apart from solubility, agglomeration of nanoparticles has also been identified to affect the toxicity of nanoparticles, which also plays an intrinsic role in their solubility. Agglomeration thus refers to a phenomenon where a group of NPs aggregate via weak forces, like van der Waals or electrostatic forces [206]. It has been reported that the degree of agglomeration in nanoparticles plays a crucial part in the distribution in the living tissues, exposure, and uptake, thus influencing the observed toxicity of NPs [207,208]. Different factors have been thought to affect the agglomeration of nanoparticles in solution, including size, surface structure, chemical composition, and shape [209,210,211]. Furthermore, its occurrence highly depends on parameters such as pH, temperature, and solution chemistry [209,212,213]. Large agglomerate of TiO_2_, according to a study, has been reported to cause a strong toxicity response than small agglomerates for glutathione depletion, IL-8 and IL-1β increase, and DNA damage in THP-1. It was concluded in this study that agglomeration influences their toxicity/biological responses, and large agglomerates do not appear less active than small agglomerates [214].

Although sufficient data are not yet available that can sufficiently paint the picture of the toxicity profile of most nanoparticles over a long period, organic nanomaterials derived from lipids, starch, protein, and chitosan have been suggested to be non-toxic, seeing that they are wholly digested are not bio-persistent in the gastrointestinal tract system of humans [215]. This, however, does not exclude them from potentially bringing harm. The large-surface-area-to-volume ratio has thus been implicated as a possible concern due to increased bioavailability brought about by the size [44]. This, therefore, suggest the importance of both in vivo and in vitro studies in ascertaining their safety for humans. Similarly, testing to determine the level of migration of nanoparticles into food substances is essential in ensuring the use of metal-based material in food packaging. For instance, some reports have already examined the migration of silver NPs into food products and have found that, although silver plays various significant roles in the design of packaging materials, it can cause genotoxicity and neurotoxicity [195]. Silver NPs have been reported to get deposited in the kidney, liver, testicles, and brain even though their migration in food is very low due to the low concentrations often applied [216]. Other reports have suggested that hydrophilic nanoparticles that are positively charged possess the ability to increase blood circulation in an intense manner which can lead to organ compromise. Still, these reports have been thought to need further verifications [217]. Despite the various concerns surrounding the use of this material, reports have also been made to suggest that if the nanoparticles are adequately embedded in the matrix of the used polymeric materials, migration may be significantly reduced. However, external factors may still bring about their migration into food materials. Hence, in applying nanomaterial, especially metal-based ones, into food packaging material, it is crucial to conduct studies that ascertain their toxicity, migrations, permissible limits, and their interaction with polymer matrices before they can be applied as food packing material [44]. In other words, every engineered nano-based material should be scrutinized, from manufacturing to storage and distribution to disposal.

Furthermore, there is a need for continued exploration of the cause and mechanism of nanotoxicity in other to gain adequate knowledge and understanding [192]. Adequate and deliberate laws and policies relating to the manufacturing, application, and recycling of nanomaterials should be set to avoid any concerns that may arise in their applications [192]. Consequently, the FDA, and other international bodies, like the EU, have provided adequate information and guidance to evaluate the safe use of nanoparticles in food packaging alongside standardized procedures to analyze the risk to humans and the environment [191].

## 5. Conclusions

Nanotechnology has provided a plethora of user-friendly alternative platforms to researchers in different field of endeavor, including agriculture. Its promising solutions in improving agricultural productivity and reducing losses have made this technology highly sought after. This technology, likewise, has benefited industrial food processing sectors with enhanced food production, excellent market value, high nutritional and sensing properties, improved safety, and better antimicrobial protection. This has led to its wide application in recent years in post-harvest technology. Specifically, its application in food packaging has been on the rise owing to the vast ease of preparation, alongside the accompanying useful physicochemical and biological properties. Metal-based nanoparticles, especially Ag, ZnO, TiO_2_ and CuO, have generally been at the forefront of these applications due to their ability to nicely integrate into different polymer matrices, including biopolymers, which confers a superior property. Hence, metal-based nanoparticles, in the design of functional food-packaging material, have been found to confer different biological properties such as antioxidant, antimicrobial, and anti-inflammatory activities while still enhancing the mechanical, physical, barrier and optical properties of the base material. Although many concerns have been raised regarding their continuous usage in food and drugs, owing to toxicity and bioaccumulation via migration, adequate guidance alongside standardized procedures for analyzing the risk-benefit index in humans and the environment are currently being explored and discussed. Metal-based nanoparticles thus offer a promising platform in food packaging technology if the issues regarding toxicity are carefully and deliberately allayed through other technological approaches.

## Figures and Tables

**Table 1:** Examples of some commercially available nano-based food packaging materials and their functions. (Extracted and modified from [44]).

Material Type	Form	Application Product	Function	Brand and Company
Nylon 6-nanoclay composite	Barrier nylon resins	Beer and flavoured alcoholic beverage bottles, PET	Oxygen scavenging	Aegis HFX Resin and OXCE Resin. Honeywell International Inc., Phoenix, AZ, USA
Iron Oxidation	Sachets & Film	Fried snacks	Oxygen scavenger	OxyGuard®, Clariant Ltd., Mutten, Swaziland
Allyl isothiocyanate (AIT) or scavenging molecular O_2_ (Listeria populations)	Tray/Pads	Ham, ready-to-eat meat product	CO_2_ emitter and antimicrobial pad	UltraZap R Xtenda Pak pads. Paper Pak Industries, Winnipeg, MB, Canada
Titanium Dioxide (Nanoencapsulation)	Powder	Powdered milk-based products	Anticaking	Carnation Instant food. Carnation Breakfast Essential, Vevey, Switzerland
Nanosilver	Bag, Spray	Fruits & Vegetables	Antimicrobial actions	Biomaster. Addmaster Limited, Monrovia, CA, USA
Nanoclay Al_2_O_3_ • 2SiO_2_ • 2H_2_O	Film	Dried Fruits, cheeses, Coffee	Gas Barrier	N-coat. Multifilm Packing Corporation, Elgin, IL, USA
Changing colour based on aromatic compounds (sensor)	stickers	Fruits	Freshness Indicators	RipeSense™. Ripesense Limited, Tauranga, New Zealand.
Cerium oxide	Film	Retort Products and hot fill of meat and fish products	Oxygen scavenger	OMAC® Imperm®. Mitsubishi Gas Chemical Inc., Chiyoda-ku, Japan
sodium carbonate/sodium glycinate	Sachets/ Labels	Strawberries, eggplant	CO_2_ scavenger	Ageless®. Mitsubishi Gas Chemical Inc., Chiyoda-ku, Japan
TTI based on enzyme, Lipase, and pH indicating dye	Stickers	Seafood, Oysters	Freshness (Based on color)	TimeStrip®. TimeStrip UK Ltd., Cambridge, UK.

**Table 2:** Some examples of natural biopolymers with different metal-based nanoparticles and their role in the design of some active food packaging materials. Adapted and modified from [58], with permission from Enviro Research Publishers.

Metal-Based Nanoparticles Enmeshed in Some Natural Biopolymers	Observed Properties Due to the Metal-Based Nanoparticles
Carboxymethyl cellulose/ZnO, CuO, and Ag	Enhanced rate of UV absorption. Decreased water vapor permeability (WVP). Improvement of Young’s modulus (YM), tensile strength (TS), and elongation at break (EB). [66]
Starch/Ag-ZnO-CuO	Decreased water solubility (WS), water vapor permeability (WVP), and elongation at break (EB). Increase of TS and YM. Optimum UV and visible absorbance. [67]
Fish skin gelatin/Ag-Cu	Enhanced thickness, TS, a* (red/green) and b* (yellow/blue) in value, total colour difference (ΔE), transparency, and thermal degradation temperature (TDT). Decreased EB and lightness (L*) value. Darker colour. [68]
Gelatin-starch/ZnO	Enhanced thickness, TS, and melting temperature. Decreased EB, WVP, and WS. [69]
Soy protein/ZnO	Decreased L* value, whiteness index, and OTR (oxygen transmission rate). Enhanced a* and b* value, transparency, ΔE, EB, and TS. Showed good barrier properties against UV and visible light. WVP isolate. [70]
Gelatin-PVA/TiO_2_-ZnO	Decreased oxygen permeability (OP), transparency, WVP, and EB. Enhanced YM, TS, and thickness. [71]
CMC-chitosan/ZnO	Decreased a*, b*, and L* value, YM, and TS. Enhanced b* value, chroma value, ∆E, EB, and contact angle reduction. [72]
Galactomannan/ZnO	Enhanced contact angle, TS, TDT, YM, UVA, and UVB absorption. Decreased OP and WVP. [73]
Nanolignin-PLLA/Ag, Ag_2_O, TiO_2_, WO_3_, Fe_2_O_3_ and ZnFe_2_O	Enhanced a* and b* value, TS, thickness, ∆E, and YM. Decreased EB, WVP, and L* value. [74]
Poly(3-hydroxybutyrate-co-3-hydroxyvalerate)/ZnO	Decreased a*, b*, and L* value and EB. Enhanced TS, TDT, transparency, and toughness. [75]

UVA and UVB: types of UV radiation.

**Table 3:** Selected food packaging materials containing silver nanoparticles.

Polymer Matrix	Quantity/Percentage (%) Weight of Ag	Notable Properties	References
liposomes were used to encapsulate Laurel essential oil (LEO) and AgNPs, mixed with chitosan to coat polyethylene (PE) films. (PC-Lip/LEO/AgNPs)	Not indicated	PC-Lip/LEO/AgNPs films showed good useful antioxidant and antimicrobial properties. Its evaluation on pork meat showed the extension of shelf life from 9 days, which is for pure PE, to 15 days without cytotoxicity.	[101]
polylactic acid (PLA)/AgNPs	1–10%	Preserved ascorbic acid in strawberries. Decreased the reduction rate of polyphenols in the same fruit. PLA/Ag 5% film showed better preservative properties than the other counterparts.	[102]
Polyvinyl alcohol/clay/AgNPs nanocomposites film	Not indicated	Enhanced mechanical, light barrier and water-resistant properties were observed. Antimicrobial action against *S. Typhimurium* and *S. aureus* enabled it as active food packaging material. Fabricated Pouches of PVA/clay/Ag nanocomposite prevented microbial spoilage in chicken sausages.	[103]
Polyethylene/Ag/TiO_2_	Ag/TiO2 nanopowder (9 g)	Showed strong antibacterial activity because of the interaction between Ag and TiO_2_. This film retarded the changes in the pasting qualities and texture of rice.	[104]
(AgNPs) encapsulated in gelatin-montmorillonite(M), cellulose acetate (CA), and/or thymol. CA/Ag/M film	3–5%	tensile properties, UV blocking, and oxygen barrier properties of the films were enhanced. Good antioxidant activity was recorded, including those having thymol. Synergistic effects of AgNPs and thymol on the films’ antimicrobial and antifungal activities.	[105]

**Table 4:** Selected studies involving the incorporation of ZnO NPs in biocomposite materials for food packaging applications.

Polymer Matrix	Concentration/Percentage (%) Weight of ZnO	Notable Properties	References
Carboxymethyl cellulose (CMC)/curcumin/ZnO functional film	1.0%	2,2′-Azino-bis(3-ethylbenzothiazoline-6-sulfonic acid (ABTS) value increased to 7.5% and 92.5% upon the introduction of 1% of ZnO NPs and curcumin to the film, respectively. Improved UV barrier and mechanical properties upon the addition of 0.5 wt% of curcumin and 1 wt% of ZnO. CMC film with of 1 wt% ZnO and curcumin (CMC/Cur1.0/ZnO1.0) showed optimal functional films with antibacterial and antioxidant properties.	[117]
Bacteria cellulose (BC) *Gluconacetobacter xylinum*/ZnO (mediated propolis extract) Functional films	Not indicated	Showed antimicrobial properties with a minimum inhibition concentration (MIC) recorded at 0.438 mg/mL on *Escherichia coli, Bacillus subtilis*, and *Candida albicans* upon the addition of ZnO on the bacteria cellulose matrix.	[118]
Gelatin/starch/ZnO composite films	12.5% of ZnO was used in preparing the nanocomposite	Incorporating ZnO enhanced the tensile strength (0.20–0.22 MPa) and reduced the elongation at breaks and film solubility. The bio-nanocomposite films showed antibacterial properties against *Staphylococcus aureus* and *Escherichia coli.*	[69]
Starch/polyvinyl alcohol (PVA)/ZnO composite films	3.12 μg/mL (Minimum inhibitory concentration value was used)	Enhanced water barrier, UV barrier, mechanical and antimicrobial properties. In the antimicrobial study, an inhibition zone of 28 mm was recorded against *Salmonella typhimurium*.	[119]
Grape seed extract (GSE, 5 wt% of CMC)/ZnO composite films	3%	The inclusion of GSE enhanced antioxidant activity to the CMC-based films, exhibiting about 95% and 25% scavenging activity against ABTS and DPPH oxidative free radicals. The film exhibited 100% UV protection. Furthermore, upon the addition of ZnO NPs, the composite film showed enhanced mechanical and water vapor barrier properties. Also, the composite film displayed potent antibacterial properties against foodborne pathogens of *E. coli* and *L. monocytogenes*.	[120]
Pectin/ZnO composite films	0.5–1.5%	The UV-light barrier property of the pectin/ZnO films was significantly enhanced as the concentration of ZnO increased.	[121]

**Table 5:** Few examples of TiO_2_ NPs-based materials and their effects on the polymer matrix [122].

Polymer Matrix	Percentage (%) Weight of TiO_2_ in the Total Mixture of the Composite	Effects/Functions of TiO_2_ in the Prepared Food Packaging Material	References
Sodium caseinate/guar gum	1–2	Enhanced tensile strength and antimicrobial properties; reduced the permeability of water vapor and solubility.	[140]
Alginate and Aloe vera gel	1–5	Enhanced tensile strength, opacity, elongation-at-break, and antibacterial properties; Reduced water vapor permeability.	[141]
Starch/poly(vinyl alcohol)	0.01–1	Enhanced tensile strength and opacity; Reduced water vapor permeability.	[142]
Gelatin	0.5–2	Enhanced opacity. Reduced the water vapor permeability and water content.	[143]
Chitosan	1	Enhanced tensile strength and shelf life; reduced the permeability of water vapor.	[144]
Gelatin	0.5–5	Enhanced tensile strength and antimicrobial properties; reduced the permeability of water vapor.	[145]
Sago starch	1–5	Enhanced tensile strength, opacity, and antibacterial properties. Reduced the water vapor permeability, water content, and water solubility.	[146]
Whey protein isolate	0.1–2	Enhanced tensile strength, opacity, elongation-at-break, Reduced water content, and water solubility.	[147]
Hydroxypropyl methylcellulose	0.5–2	Enhanced opacity and elongation-at-break (EB).	[143]
k-carrageenan/xanthan gum/ gellan gum	1–7	Enhanced tensile strength and antimicrobial properties; reduced water vapor permeability and water content.	[139]
Gelatin	3–5	Enhanced tensile strength, opacity, elongation-at-break, and antibacterial properties; Reduced water vapor permeability.	[148]
Sweet potato starch/lemon- waste pectin	0.5–4	Enhanced tensile strength. Reduced the water vapor permeability, water content, and water solubility.	[149]
Gellan gum	1–20	Enhanced thickness, tensile strength, opacity, and antimicrobial properties; reduced the permeability of water vapor.	[150]
Hydroxypropyl methylcellulose	0.04	Enhanced elongation-at-break.	[151]
Chitosan	0.25–2	Enhanced tensile strength and antimicrobial properties; reduced the permeability of water vapor.	[152]
Kefiran/whey protein isolate	1–5	Enhanced elongation-at-break. Reduced the water vapor permeability, water content, and water solubility.	[131]
CMC/guanidinylatedchitosan	1–5	Enhanced tensile strength, opacity, and antibacterial properties. Reduced the water vapor permeability, water content, and water solubility.	[153]
Wheat starch	1–4	Enhanced opacity. Reduced the water vapor permeability and water solubility.	[154]

**Table 6:** Some studies showing the effect of ZnO nanoparticles with some polysaccharides on different fruits. Table adapted and modified from [177]. Adapted from [177], with permission from MDPI.

Polysaccharide Materials and ZnO Quantity	Study Conditions	Effect of Coating	References
5% *w*/*v* of Chitosan 1%. ZnO *v*/*v* gel.	Dipping Stored at 21 ± 1 °C for 20 days and 80% RH Effect Guava	Reduced colour change and weight loss and maintained firmness. Ripening ratio index (SS/TA) was reduced. No external injuries were observed until end of storage.	[178]
5% *w*/*v* of Alginate. ZnO 1% *w*/*v* gel.	Dipping 20 days at 21 ± 1 °C and RH 80 Guava	None	[178]
5% *w*/*v* of Alginate–chitosan (90–10%). ZnO 1% *w*/*v* gel.	Dipping 20 days at 21 ± 1 °C and RH 80 Guava	Maintained firmness better and prevented external injuries.	[178]
3 g of Chitosan in 0.4 L coating solution. ZnO at varying percentages between 0.005–0.027% *w*/*w* coating solution (611.30 nm).	Dipping 12 days, 10 °C Fresh-cut papaya	Microbial growth/action was reduced.	[179]
1.5% *w*/*v* of Alginate. ZnO at varying concentrations between 0.25 and 1.25 g/L (30–50 nm).	Dipping 20 days, 1 °C, RH 95% Strawberry	Reduced colour change, weight loss, and maintain firmness. Increases antimicrobial and antioxidant activity by maintaining ascorbic acid. Control fruit maturity, improve Titrable acidity (TA) and prevent increase of Total soluble solids (TSS). Reduces the decrease in anthocyanin, phenolic, and peroxidase activity and decreases superoxide dismutase activity. Extended the storage life of fresh fruits by up to 20 days	[180]
0.8 g of Carrageenan in 0.1 L solution. 1% *w*/*w* of carrageenan + ZnO 0.5%.	Dipping 20 °C and RH 61% Mango	Reduced the production of CO_2_ and weight loss. Maintained total acidity, colour, and textural appearance better.	[181]
0.5% *w*/*v* of CMC. ZnO 0.1% and 0.2% *w*/*v* (30–100 nm).	Dipping 12 days, 4 °C and RH 90% Pomegranate arils	Reduced weight loss and loss of vitamin C. Reduced the loss of anthocyanin and phenolic content. Showed higher antioxidant activities	[182]
10 g of Pectin in 1 L solution. ZnO 0.1 g/L in solution	Dipping 8 days at 25 °C Star fruit	Reduced browning index, redness value, and weight loss. Reduced physical damage.	[183]

## Data Availability

Not applicable.
